# The Influence of Resiniferatoxin (RTX) and Tetrodotoxin (TTX) on the Distribution, Relative Frequency, and Chemical Coding of Noradrenergic and Cholinergic Nerve Fibers Supplying the Porcine Urinary Bladder Wall

**DOI:** 10.3390/toxins9100310

**Published:** 2017-10-03

**Authors:** Ewa Lepiarczyk, Agnieszka Bossowska, Jerzy Kaleczyc, Agnieszka Skowrońska, Marta Majewska, Michal Majewski, Mariusz Majewski

**Affiliations:** 1 Department of Human Physiology, Faculty of Medical Sciences, University of Warmia and Mazury in Olsztyn, Warszawska 30, 10-082 Olsztyn, Poland; agnboss@uwm.edu.pl (A.B.); agnieszka.skowronska@uwm.edu.pl (A.S.); marta.majewska@uwm.edu.pl (M.M.); mariusz.majewski@uwm.edu.pl (M.M.); 2 Department of Animal Anatomy, Faculty of Veterinary Medicine, University of Warmia and Mazury in Olsztyn, 10-719 Olsztyn, Poland; jerzy.kaleczyc@uwm.edu.pl; 3 Department of Pharmacology and Toxicology, Faculty of Medical Sciences, University of Warmia and Mazury, Warszawska 30, 10-082 Olsztyn, Poland; michal.majewski@uwm.edu.pl

**Keywords:** resiniferatoxin, tetrodotoxin, nerve fibers, urinary bladder, immunohistochemistry, pig

## Abstract

The present study investigated the influence of intravesically instilled resiniferatoxin (RTX) or tetrodotoxin (TTX) on the distribution, number, and chemical coding of noradrenergic and cholinergic nerve fibers (NF) supplying the urinary bladder in female pigs. Samples from the bladder wall were processed for double-labelling immunofluorescence with antibodies against cholinergic and noradrenergic markers and some other neurotransmitter substances. Both RTX and TTX caused a significant decrease in the number of cholinergic NF in the urinary bladder wall (in the muscle coat, submucosa, and beneath the urothelium). RTX instillation resulted in a decrease in the number of noradrenergic NF in the submucosa and urothelium, while TTX treatment caused a significant increase in the number of these axons in all the layers. The most remarkable changes in the chemical coding of the NF comprised a distinct decrease in the number of the cholinergic NF immunoreactive to CGRP (calcitonin gene-related peptide), nNOS (neuronal nitric oxide synthase), SOM (somatostatin) or VIP (vasoactive intestinal polypeptide), and an increase in the number of noradrenergic NF immunopositive to GAL (galanin) or nNOS, both after RTX or TTX instillation. The present study is the first to suggest that both RTX and TTX can modify the number of noradrenergic and cholinergic NF supplying the porcine urinary bladder.

## 1. Introduction

Overactive bladder (OAB) is a condition characterized by the presence of urinary urgency, typically accompanied by nocturia and frequency, in the absence of urinary tract infection or another evident pathology. This disease is highly prevalent, especially among women, and significantly reduces patient’s quality of life [1]. It is believed that OAB symptoms result from sudden, inappropriate contractions of the muscle in the wall of the bladder during the filling phase of the micturition cycle. Since normal and abnormal contractions of the bladder are mediated by stimulation of muscarinic receptors by acetylcholine (ACh) released from parasympathetic/cholinergic neurons supplying the organ (for review see: [2]), antimuscarinic drugs are the treatment of choice in OAB. Clinically, however, their use is often unsatisfactory because of many associating side effects, such as dry mouth, cognitive changes, constipation, urinary retention, or blurred vision, resulting from the widespread blockade of cholinergic activity [3]. Therefore, new therapeutic agents, such as neurotoxins, are being investigated for the therapy of OAB. Currently, the most well-known neurotoxins, which have been successfully applied in urology, are botulinum toxin (BTX) and resiniferatoxin (RTX) (for review see: [4]).

The urine storage process largely depends upon the undisturbed and coordinated control of sympathetic-parasympathetic innervation of the urinary bladder (for review see: [2]). In our previous study [5], we found that BTX strongly influences immunohistochemical characteristics of noradrenergic and cholinergic axons distributed in the porcine urinary bladder wall. The present contribution was aimed at comparing the influence of two other neurotoxins, RTX and tetrodotoxin (TTX), on the distribution, relative frequency, and chemical coding of cholinergic and noradrenergic nerve fibers (NF) supplying this organ.

RTX is a capsaicin analogue produced by spurge Euphorbia resinifera, and it is thousand times stronger than tetrodotoxin. The toxin acts by inhibiting the transient receptor potential of vanilloid type 1 (TRPV1) nonspecific Ca^2+^ channels located mainly on the primary afferent sensory neurons involved in nociceptive signaling (for review see: [6]). Thus, in urology, RTX is predominantly used in patients suffering from detrusor overactivity as it weakens or even blocks an exaggerated C-fiber input-dependent sacral micturition reflex [7]. TTX is a neurotoxin generally found in the liver and ovaries of several marine organisms, for instance pufferfish. The toxin acts by blocking voltage-gated sodium channels in nerve cell membranes and, consequently, similar to RTX, is able to impair nociceptive transmission (for review see: [8,9]). Because of its mechanism of action, TTX has been considered as a potential therapeutic agent in the treatment of certain pains associated with diseases such as leprosy or rheumatoid arthritis (for review see: [9]). Moreover, TTX has been often applied in scientific studies concerning the normal and abnormal functioning of mammalian urinary bladder [10,11,12].

We decided to perform the present experiment on domestic pigs, as they are considered to be one of the best animal models used in biomedical research because of their anatomical and physiological resemblance to humans in terms of the urinary, cardiovascular, integumentary, and digestive systems [13,14,15,16].

## 2. Results

In the present study, we used two different control groups. Six pigs served as controls for RTX-instilled animals and were treated with intravesical instillation of 5% aqueous solution of ethyl alcohol (60 mL). Another six pigs served as the controls for TTX-instilled animals and they were treated with intravesical instillation of 20 mM citrate buffer (pH 4.9, 60 mL per animal). The above mentioned procedures were performed in the control pigs to ensure that changes in the chemical coding of NF after the toxin treatment (RTX or TTX, respectively) were caused by the toxins themselves, not due to factors associated with the administration processes. Nevertheless, it should be emphasized that no significant differences in the distribution and relative frequency of either vesicular acetylcholine transporter-immunoreactive (VAChT-IR) or dopamine β-hydroxylase-immunoreactive (DβH-IR) NF were found between the two control groups. Therefore, these results will be presented together and described in the next section.

### 2.1. The Distribution and Relative Frequency of VAChT-Immunoreactive Nerve Fibers

#### 2.1.1. Control Animals

**Figure 1:**
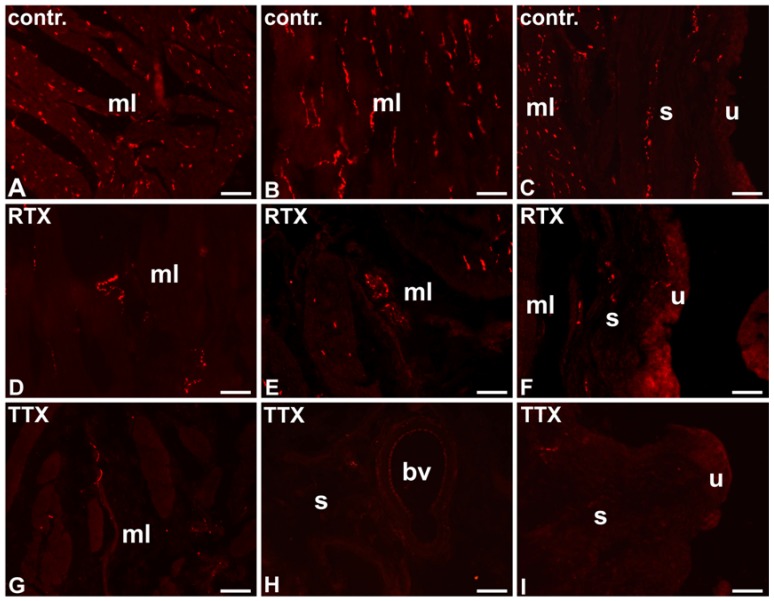
The distribution and relative frequency of VAChT-IR nerve terminals in the control (contr.; **A**–**C**), RTX-treated (RTX; **D**–**F**), or TTX-treated (TTX; **G**–**I**) pigs; muscle layer (mL), blood vessel (bv), submucosa (s), urothelium (u); 20×.

In the control pigs, the muscle layer was densely supplied with VAChT-IR NF. Many cholinergic axons were found around blood vessels. A moderate number of VAChT- IR NF was observed in the submucosa and only a few axons penetrated into the urothelium (Table 1; Figure 1A–C).

**Figure 2:**
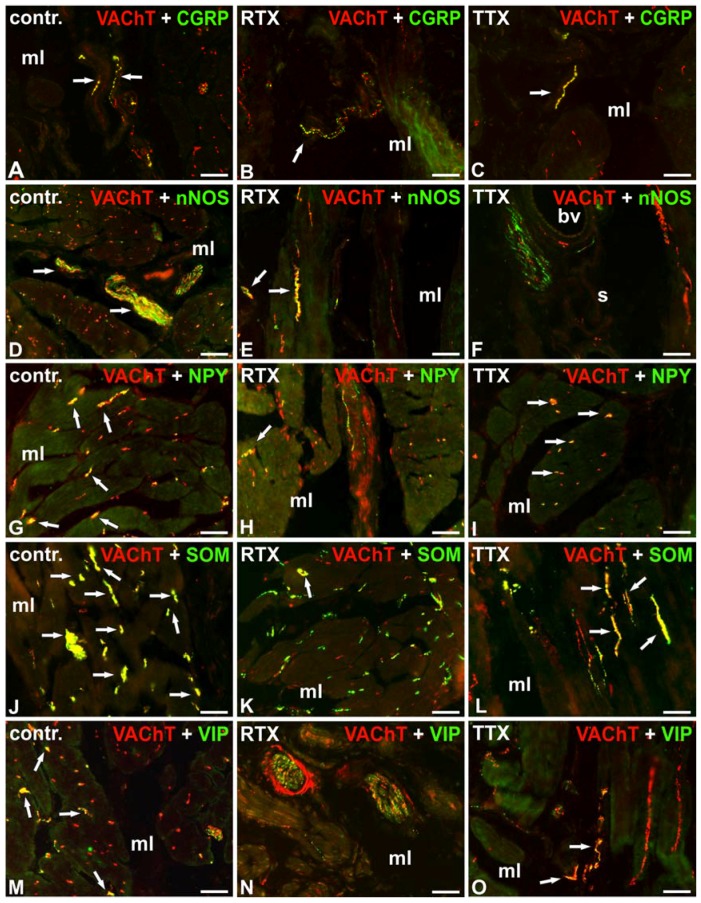
Distribution of VAChT-(red; labelled with CY3) and calcitonin gene-related peptide- (CGRP)- (**A**–**C**), neuronal nitric oxide synthase- (nNOS)- (**D**–**F**), neuropeptide Y- (NPY)- (**G**–**I**), somatostatin- (SOM)- (**J**–**L**) or vasoactive intestinal polypeptide- (VIP)- (**M**–**O**) positive (green; labelled with fluorescein isothiocyanate (FITC)) nerve fibers in the urinary bladder wall in the normal (**A**,**D**,**G**,**J**,**M**), RTX-treated (**B**,**E**,**H**,**K**,**N**), or TTX-treated (**C**,**F**,**I**,**L**,**O**) pigs. Red and green channels were digitally superimposed. Double-labelled fibers are yellow to orange, and most of them are indicated with arrows; muscle layer (mL), blood vessel (bv), submucosa (s), urothelium (u); 20×.

Most of the cholinergic NF supplying the muscle coat were immunopositive to somatostatin (SOM) and many stained for neuropeptide Y (NPY) or neuronal nitric oxide synthase (nNOS). Only single VAChT-IR NF observed in the muscle layer were immunoreactive (IR) to calcitonin gene-related peptide (CGRP) or vasoactive intestinal polypeptide (VIP). The majority of VAChT-IR NF surrounding blood vessels were immunopositive to SOM and single VAChT-IR nerve terminals revealed also immunoreactivity to CGRP, nNOS, NPY, or VIP. VAChT-IR NF observed in the submucosa were predominantly IR to SOM. A moderate number of these nerves expressed immunoreactivity to VIP and single VAChT-IR axons stained for CGRP or nNOS. Few cholinergic nerve terminals penetrating beneath the urothelium were CGRP-, SOM-, or VIP-positive. The cholinergic NF were galanin- (GAL)-, Leu5–enkephalin- (L-ENK)-, pituitary adenylate cyclase-activating polypeptide- (PACAP)- or substance P- (SP)–immunonegative (Table 2; Figure 2A,D,G,J,M).

#### 2.1.2. RTX-Treated Pigs

After RTX intravesical treatment, the number of VAChT-IR NF in the smooth muscle layer and around blood vessels was smaller than that observed in the control pigs; a moderate number of these nerve terminals were found in the smooth muscle layer, and only a few VAChT-IR nerve endings were distributed around blood vessels. As in the control group, a moderate number of cholinergic NF were distributed in the submucosa and a few axons penetrated to the urothelium (Table 1; Figure 1D–F).

Double-labeling immunofluorescence revealed that RTX treatment caused a decrease in the number of cholinergic NF immunoreactive to CGRP, nNOS, NPY, SOM, or VIP in the smooth muscle layer and around the blood vessels, as a moderate number of VAChT-IR NF were NPY or SOM-IR, and single cholinergic axons stained also for CGRP, nNOS, or VIP. RTX instillation was followed by a decrease in the number of VAChT-IR axons stained for SOM in the submucosa. Moreover, in contrast to the findings obtained in the control group, no cholinergic NF immunoreactive to CGRP or VIP were found in the submucosa and beneath the urothelium. As in the control group, the cholinergic NF were GAL-, L-ENK-, PACAP-, and SP-immunonegative (Table 2; Figure 2B,E,H,K,N).

#### 2.1.3. TTX-Treated Pigs

After TTX instillation, the number of VAChT-IR NF was smaller in all the layers of the urinary bladder wall than that observed in the control pigs; a moderate number of these axons were found in the smooth muscle layer, and single cholinergic NF were distributed in the submucosa and around blood vessels. No VAChT-IR NF were found beneath the urothelium (Table 1; Figure 1G–I).

Double-labeling immunofluorescence revealed that in the smooth muscle layer, similar to the control group, many VAChT-IR axons were also NPY-IR and few cholinergic axons stained for CGRP or VIP. Again, few cholinergic NF associated with the blood vessels were NPY-IR. However, TTX treatment caused a distinct decrease in the number of cholinergic NF immunoreactive to nNOS or SOM in all the investigated layers of the urinary bladder wall. TTX instillation was also followed by a significant decrease in the number of cholinergic NF surrounding blood vessels and revealing immunoreactivity to CGRP or VIP in the submucosa and beneath the urothelium. As in the control group, the cholinergic NF were GAL-, L-ENK-, PACAP-, and SP-immunonegative (Table 2; Figure 2C,F,I,L,O).

### 2.2. The Distribution and Relative Frequency of DβH-Immunoreactive Nerve Fibers

#### 2.2.1. Control Animals

**Figure 3:**
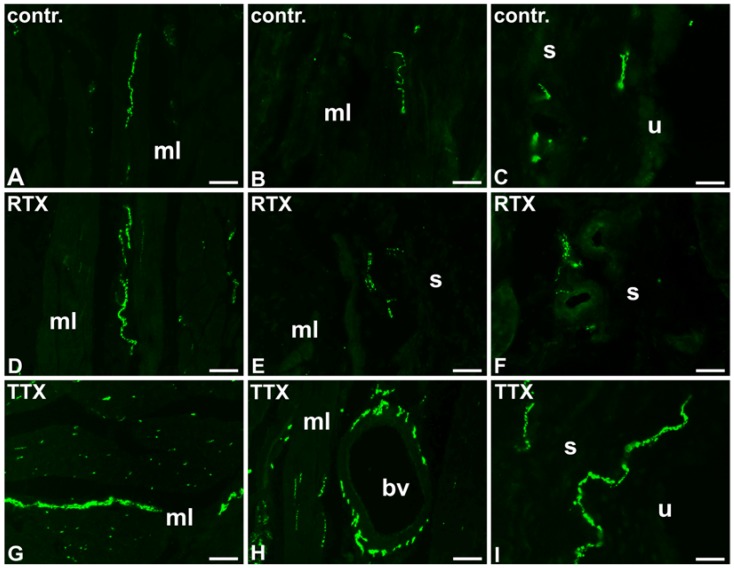
The distribution and relative frequency of DβH-immunoreactive nerve fibers in control (contr.; **A**–**C**), RTX-treated (RTX; **D**–**F**), or TTX-treated (TTX; **G**–**I**) pigs; muscle layer (mL), blood vessel (bv), submucosa (s), urothelium (u); 20×.

In the control pigs, the urinary bladder smooth muscle layer was sparsely supplied with noradrenergic NF, however, many DβH-IR axons surrounded blood vessels. A moderate number of DβH-IR NF was distributed in the submucosa and some of them penetrated beneath the urothelium (Table 3; Figure 3A–C).

**Figure 4:**
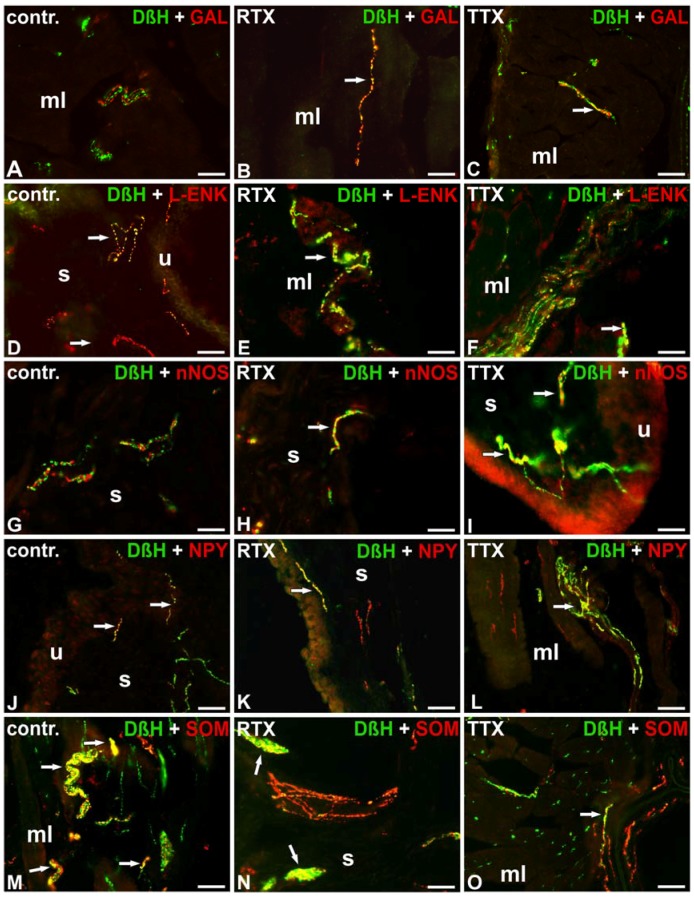
Distribution of DβH-(green; labelled with FITC) and GAL- (**A**–**C**), L-ENK- (**D**–**F**), nNOS- (**G**–**I**), NPY- (**J**–**L**) or SOM- (**M**–**O**) positive (red; labelled with CY3) nerve fibers in the urinary bladder wall in the normal (**A**,**D**,**G**,**J**,**M**), RTX-treated (**B**,**E**,**H**,**K**,**N**), or TTX-treated (**C**,**F**,**I**,**L**,**O**) pigs. Red and green channels were digitally superimposed. Double-labelled fibers are yellow to orange and most of them are indicated with arrows; muscle layer (mL), blood vessel (bv), submucosa (s), urothelium (u); 20× (**A**–**O**); 40× (**I**).

Double-labeling investigations revealed that many noradrenergic NF supplying the muscle coat were IR to NPY and a moderate number of DβH-IR axons stained for L-ENK or SOM. Solitary DβH-IR NF were immunopositive to CGRP. Most noradrenergic NF observed around blood vessels were IR to NPY, and single DβH-IR axons exhibited immunoreactivity to L-ENK or SOM. In the submucosa and beneath the urothelium, only a few DβH-IR NF were L-ENK-, NPY-, or SOM-IR. The noradrenergic NF were GAL, nNOS, PACAP, SP, or VIP-immunonegative (Table 4; Figure 4A,D,G,J,M).

#### 2.2.2. RTX-Treated Pigs

In the urinary bladder wall of pigs treated with RTX, the number of DβH-positive NF in the smooth muscle layer and around blood vessels was similar to that observed in the control pigs; a small number of DβH-IR axons were found in the smooth muscle layer, while blood vessels were very densely supplied by these NF. However, a smaller number of the NF were distributed in the submucosa as only a few axons were found there, and no axons were observed to penetrate into the urothelium (Table 3; Figure 3D–F).

The chemical profile of the noradrenergic NF after RTX instillation was generally comparable to that observed in the control pigs. However, both in the muscle coat and around the blood vessels the number of noradrenergic NF IR to GAL was slightly higher. Moreover, a lower number of DβH-IR NF containing immunoreactivity to NPY were found in the muscle layer. Unlike in the control animals, some noradrenergic axons supplying the submucosa and urothelium were nNOS-IR. The noradrenergic NF were PACAP-, SP-, and VIP-immunonegative (Table 4; Figure 4B,E,H,K,N).

#### 2.2.3. TTX-Treated Pigs

In the TTX treated pigs, the number of DβH-IR NF was significantly higher in all the layers of the urinary bladder wall than in that of the control animals; a moderate number of DβH-IR NF was distributed in the muscle coat, many of these nerve terminals were observed in the submucosa and few noradrenergic axons penetrated into the urothelium. Similar to the control pigs, blood vessels were densely supplied with DβH-IR NF (Table 3; Figure 3G–I).

Double-labeling investigations revealed some distinct changes in the chemical coding of the noradrenergic NF after RTX treatment. In the muscle layer, in contrast to the findings obtained from the control group, single DβH-IR NF revealed immunoreactivity to GAL. In the muscle layer and around the blood vessels, the number of DβH-IR nerve terminals containing immunoreactivity to L-ENK or SOM was also higher. The number of DβH/NPY-IR axons was definitely lower in all the investigated areas of the urinary bladder wall. Additionally, again, in contrast to the findings obtained from the control group, a moderate number of DβH-IR NF expressed immunoreactivity to nNOS in the submucosa and beneath the urothelium. DβH-positive NF were PACAP-, SP-, and VIP-immunonegative (Table 4; Figure 4C,F,I,L,O).

## 3. Discussion

The results of the present study clearly indicate, that application of either TTX or RTX is followed by meaningful changes in the distribution, relative frequency, and chemical coding of noradrenergic and cholinergic NF supplying the wall of the porcine urinary bladder.

A thorough discussion regarding the distribution, relative frequency, and chemical coding of noradrenergic and cholinergic NF supplying the wall of the female porcine urinary bladder was already presented in our previous paper [5]. For each of the toxins investigated, a different control group was used. In the earlier study [5], in the control pigs, no medical procedures were applied. In the present experiment, the animals included to the first control group were intravesically instilled with a 5% aqueous solution of ethyl alcohol, while the pigs assigned to the second control group were instilled with a citrate buffer. The aim of the above mentioned procedures was to guarantee that changes in the distribution and chemical coding of NF after the toxin treatment (BTX in the previous study, and RTX or TTX in the present experiment) were caused by the toxins themselves, not due to factors associated with the technique and route of their administration. It needs to be highlighted, that the number, sex, body weight, and age of the animals used as controls in both experiments as well as the surgical and immunohistochemical procedures applied were entirely corresponding. Accordingly, no significant differences in the distribution or chemical coding of NF were observed between the animals in the control groups. Therefore, in the present discussion we are concentrating on the data dealing with the changes caused by either RTX or TTX treatment.

The present findings suggest that both RTX and TTX, like BTX [5], are factors evoking very strong adaptation changes in autonomic neurons supplying the urinary bladder wall. These changes include modifications of the chemical phenotype and/or alterations in the density of NF. This seems to be an interesting finding, especially in the light of the information that RTX as well as TTX exert their primary therapeutic effect by inhibiting the noxious sensory transmission [6,8,9]. Because of their main mechanisms of action, scarce studies on the effect of either RTX or TTX on the innervation of the urinary bladder wall pertain to the influence of these toxins on sensory neurons. However, the present results suggest that the therapeutic effect observed after the toxin treatment can be a result of not only the inhibitory influence on the C-fibers but also involves changes (presumably beneficial) in the distribution and chemical coding of the cholinergic and adrenergic axons. The present data are partially supported by our previous findings, as we already have revealed that intravesically instilled RTX influences immunohistochemical characteristics of sympathetic chain ganglia urinary bladder-projecting neurons [17] and that both RTX and TTX induce plastic changes in caudal mesenteric ganglia urinary bladder-projecting neurons [18,19].

The present study suggests that both investigated toxins distinctly decrease the number of cholinergic nerve terminals in all the layers of the urinary bladder wall. Interestingly, in this respect, they seem to act similarly to BTX [5]. As was mentioned in the introduction section, anticholinergic agents are first-line pharmacotherapy for OAB because of their ability to reduce bladder contractility. Therefore, the anticholinergic effect observed after the application of the investigated toxins may suggest that their mode of action may be based on more than just the ability to block an exaggerated sensory C-fiber input from the urinary bladder wall. This bidirectional action of the investigated toxins should be considered as an advantageous effect as it has been found that antimuscarinic agents which decrease urine volume through C-fibers in the bladder are beneficial for treatment of urinary bladder disorders such as nocturia with nocturnal polyuria [20].

Furthermore, the present study has revealed that both RTX and TTX induce changes in the chemical phenotype of the investigated cholinergic nerve terminals, as the treatment with either of the toxins was followed by a decrease in the number of VAChT-IR NF that were immunopositive to CGRP, nNOS, SOM, or VIP. Additionally, RTX intravesical instillation, in contrast to TTX instillation, resulted in a decrease in the number of VAChT-IR/NPY-IR NF. The reason for the decreased expression of some of the investigated neurotransmitters or their markers in the cholinergic axons after the toxins instillation is not clear, as their functions when co-expressed with ACh are still not fully understood in the urinary tract. However, it may be expected that all these substances somehow influence the contractility of the bladder’s smooth musculature.

The toxins investigated in the present study act differently on the distribution and relative frequency of DβH-IR NF. RTX seems to have little impact on the noradrenergic innervation of the urinary bladder wall (only a small decrease in the number of DβH-IR axons was observed in the submucosal layer and beneath the urothelium). TTX, on the other hand, visibly increases the number of DβH-IR NF in the muscle layer, submucosal layer, and beneath the urothelium. In this regard, TTX seems to exert a similar effect to that of BTX, as it has been found that intravesical injections of BTX are also followed by an increase in the number of noradrenergic nerve terminals in the porcine urinary bladder wall [5]. The function of sympathetic innervation of the urinary bladder is opposite to that of parasympathetic innervation. The sympathetic neurotransmitter norepinephrine (NA) inhibits the detrusor muscle by β3-adrenergic receptors and leads to a tonicization of the bladder neck and the smooth-muscular urethra by α-adrenergic receptors, thus ensuring continence (for review see: [21]). For that reason, except of anticholinergic drugs, one of the treatments approved for use in OAB includes the β3-receptor agonists [21]. Therefore, the observed increase in the number of noradrenergic NF after TTX application could be a factor which additionally decreases the spasticity of the overreactive bladder and thus improves the therapy. The present study indicates that both RTX and TTX intravesical instillations induce a decrease in the number of noradrenergic nerve terminals immunopositive to NPY. Additionally, TTX intravesical instillation, in contrast to RTX instillation, was followed by an increase in the number of DβH-IR/L-ENK-IR or DβH-IR/SOM-IR NF. Surprisingly, after both RTX or TTX treatment, some noradrenergic nerve terminals in the urinary bladder wall revealed immunofluorescence to either GAL or nNOS, while such colocalization was not observed in the control animals. Interestingly, both GAL and nitric oxide (NO) seem to exert an inhibitory role in the micturition reflex. It has been found, that galanin delays the onset of micturition through activation of the opioid mechanism [22] while NO relaxes isolated urinary bladder smooth muscle preparations (for review see: [23]).

In conclusion, the present study has revealed the existence of profound differences in the distribution, relative frequency, and chemical coding of cholinergic and noradrenergic NF supplying the wall of urinary bladders in normal female pigs and in female pigs after intravesical RTX or TTX injections. Therefore, it should be assumed that the therapeutic effects of either of this toxins on the mammalian urinary bladder can be partly mediated by the autonomic innervation of this organ.

## 4. Materials and Methods

### 4.1. Laboratory Animals

According to the guidelines of the Local Ethics Committee for Animal Experimentation in Olsztyn (affiliated to the National Ethics Commission for Animal Experimentation, Polish Ministry of Science and Higher Education; decision No. 94/2011 from 23 November 2011), the study was carried out on 24 female pigs (8–12 weeks old, 15–20 kg body weight, b.w.) of the Large White Polish race. The animals were kept under standard laboratory conditions. They were fed standard fodder (Grower Plus, Wipasz, Wadąg, Poland) and had free access to water.

Before performing intravesical instillations, all the pigs were pretreated with atropine (Polfa, Warsaw, Poland, 0.04 mg/kg b.w., s.c.) and azaperone (Stresnil, Janssen Pharmaceutica, Belgium, 0.5 mg/kg b.w., i.m.). Thirty minutes later, sodium pentobarbital (Tiopental, 0.5 g per animal) was given intravenously in a slow, fractionated infusion.

The pigs were assigned into four groups. Six animals served as controls for RTX-instilled animals and were treated with an intravesical instillation of a 5% aqueous solution of ethyl alcohol (60 mL). Another six pigs served as controls for TTX-administered animals, and they were treated with an intravesical instillation of a 20 mM citrate buffer pH 4.9 (60 mL per animal). A further group of six pigs was treated with RTX by an intravesical instillation of the toxin (500 nmol per animal in 60 mL of 5% aqueous solution of ethyl alcohol) in order to mimic the route of its administration practiced in humans. The last group of six pigs was treated with an intravesical instillation of TTX (12 μg of TTX dissolved in 60 mL of 20 mM citrate buffer, pH 4.9). In the case of all the intravesical instillations, 10 min after the infusion, the contents of the bladder were evacuated and the catheter was removed.

One week after the administration of the 5% aqueous solution of ethyl alcohol, citrate buffer, RTX, or TTX, all the pigs were deeply anaesthetized with sodium pentobarbital and transcardially perfused with 4% buffered paraformaldehyde (pH 7.4). All the urinary bladders were collected, postfixed in the same fixative (10 min at room temperature), washed several times in 0.1 M phosphate buffer (pH 7.4), and stored in 18% buffered sucrose (pH 7.4) at 4 °C until sectioning.

### 4.2. Sectioning of the Urinary Bladder Wall Samples and Immunohistochemical Procedure

The tissue samples analyzed in the present experiment were collected (taken) from the trigone region of the urinary bladder wall. Ten-micrometer-thick cryostat sections of the samples were processed for double-labelling immunofluorescence (according to an earlier described method [24]), using antibodies (listed in Table 5) against VAChT (marker of cholinergic fibers), DβH (marker of noradrenergic fibers), CGRP, GAL, L-ENK, nNOS, NPY, PACAP, SOM, SP, or VIP. The application of antisera raised in different species allowed investigation of the coexistence of VAChT or DβH with other substances. Each mixture of primary antibodies applied contained VAChT-antiserum or DβH-antiserum and the antiserum against one of the other biologically active substances mentioned.

The labeled sections were viewed under an Olympus BX51 microscope equipped with epi-fluorescence and an appropriate filter set for CY3-conjugated streptavidin and fluorescein isothiocyanate (FITC). The images were taken with an Olympus XM10 digital camera (Tokyo, Japan). The microscope was equipped with cellSens Dimension 1.7 Image Processing software (Olympus Soft Imaging Solutions, Münster, Germany). The distribution and relative frequencies of labeled NF were assessed semi-quantitatively [25,26] in 10 sections per one animal (5 fields per section). The evaluation of these structures in the same preparations was performed independently by two investigators (number of the NF immunoreactive to each substance was evaluated subjectively, based on a scale from - (when the NF were not found) to ++++ (a very dense meshwork of fibers).

### 4.3. Control of Specificity of the Immunohistochemical Procedures

The specificity of the staining reaction was verified by preincubation tests performed on the sections from the urinary bladder wall. Overnight preincubation of 1 mL of the primary antiserum at working dilution with 20 μg/mL of the respective peptide completely eliminated the immunoreaction (Table 6). No detectable fluorescence was exhibited by the specimens after omission and replacement of the respective primary antiserum with the corresponding non-immune serum.

## Figures and Tables

**Table 1:** The distribution and relative frequency of vesicular acetylcholine transporter-immunoreactive (VAChT-IR) nerve fibers supplying the porcine urinary bladder wall. RTX = resiniferatoxin (RTX); TTX = tetrodotoxin; − nerve fibers not found; +/− single fibers; + few fibers; ++ moderate number of fibers; +++ many fibers; ++++ a very dense meshwork of fibers; ↓ a decrease in the nerve fibers density.

Part of the Urinary Bladder Wall	Control Pigs	RTX-Treated Pigs	TTX-Treated Pigs
Muscle layer	++++	++ ↓	++ ↓
Submucosa	++	++	+/− ↓
Urothelium	+	+	− ↓
Blood vessels	+++	+ ↓	+/− ↓

**Table 2:** The degree of colocalization of VAChT with other immunoreactive substances within the nerve fibers supplying the urinary bladder wall. mL—muscle layer; bv—blood vessels; s – submucosa; u – urothelium; − nerve fibers not found; +/− single fibers; + few fibers; ++ moderate number of fibers; +++ many fibers; ++++ a very dense meshwork of fibers. GAL= galanin; L-ENK = Leu5–enkephalin; PACAP = pituitary adenylate cyclase-activating polypeptide; SP = substance P; ↓ a decrease in the nerve fibers density.

Substances	Control Pigs				RTX-Treated Pigs				TTX-Treated Pigs			
	mL	bv	s	u	mL	bv	s	u	mL	bv	s	u
VAChT/CGRP	+	+	+	+	+/− ↓	- ↓	− ↓	− ↓	+	− ↓	− ↓	− ↓
VAChT/GAL	-	-	-	-	-	-	-	-	-	-	-	-
VAChT/L-ENK	-	-	-	-	-	-	-	-	-	-	-	-
VAChT/nNOS	+++	+	+	-	+ ↓	+/− ↓	-	-	− ↓	− ↓	− ↓	-
VAChT/NPY	+++	+	-	-	++ ↓	+/− ↓	-	-	+++	+	-	-
VAChT/PACAP	-	-	-	-	-	-	-	-	-	-	-	-
VAChT/SOM	++++	++++	++++	+	++ ↓	+/− ↓	+ ↓	-	++ ↓	+ ↓	++ ↓	+/− ↓
VAChT/SP	-	-	-	-	-	-	-	-	-	-	-	-
VAChT/VIP	+	+	++	+	+/− ↓	− ↓	− ↓	− ↓	+	− ↓	+ ↓	− ↓

**Table 3:** The distribution and relative frequency of dopamine β-hydroxylase-immunoreactive (DβH-IR) nerve fibers supplying the porcine urinary bladder wall; − nerve fibers not found; +/− single fibers; + few fibers; ++ moderate number of fibers; +++ many fibers; ++++ a very dense meshwork of fibers; ↓ a decrease in the nerve fibers density; ↑ an increase in the nerve fibers density.

Part of the Urinary Bladder Wall	Control Pigs	RTX-Treated Pigs	TTX-Treated Pigs
Muscle layer	+	+	++ ↑
Submucosa	++	+ ↓	+++ ↑
Urothelium	+/−	− ↓	+ ↑
Blood vessels	++++	++++	++++

**Table 4:** The degree of colocalization of DβH with other immunoreactive substances within the nerve fibers supplying the urinary bladder wall. Muscle layer (mL); blood vessels (bv); submucosa (s); urothelium (u); − nerve fibers not found; +/− single fibers; + few fibers; ++ moderate number of fibers; +++ many fibers; ++++ a very dense meshwork of fibers. ↓ a decrease in the nerve fibers density; ↑ an increase in the nerve fibers density.

Substances	Control Pigs				RTX-Treated Pigs				TTX-Treated Pigs			
	mL	bv	s	u	mL	bv	s	u	mL	bv	s	u
DβH/CGRP	+	-	-	-	+	-	-	-	+	-	-	-
DβH/GAL	-	-	-	-	+ ↑	+ ↑	-	-	+ ↑	-	-	-
DβH/L-ENK	++	+/−	+	+	++	+/−	+	+	+++ ↑	++ ↑	+	+
DβH/nNOS	-	-	-	-	-	-	+ ↑	+ ↑	-	-	++ ↑	++ ↑
DβH/NPY	++++	++++	+	+	++ ↓	+ ↓	+/− ↓	− ↓	++ ↓	++ ↓	+/− ↓	− ↓
DβH/PACAP	-	-	-	-	-	-	-	-	-	-	-	-
DβH/SOM	++	+/−	+	+	++	+/−	+	+	+++ ↑	+ ↑	+	+
DβH/SP	-	-	-	-	-	-	-	-	-	-	-	-
DβH/VIP	-	-	-	-	-	-	-	-	-	-	-	-

**Table 5:** List of primary antisera and secondary reagents used in the study (CGRP = calcitonin gene-related peptide, DβH = dopamine β-hydroxylase, GAL = galanin, L-ENK = Leu5–enkephalin, nNOS = neuronal nitric oxide synthase, NPY = neuropeptide Y, PACAP = pituitary adenylate cyclase-activating polypeptide, SOM = somatostatin, SP = substance P, VAChT = vesicular acetylcholine transporter, VIP = vasoactive intestinal polypeptide, FITC = fluorescein isothiocyanate.

Antigen	Code	Dilution	Species	Supplier
Primary antibodies				
CGRP	T-5027	1:400	Guinea pig	Peninsula; San Carlos; CA; USA
	AB5920	1:8000	Rabbit	Millipore; Temecula; CA; USA
DβH	MAB 308	1:300	Mouse	Millipore; Temecula; CA; USA
	D9010-07A.50	1:4000	Rabbit	Biomol; Hamburg; Germany
GAL	T-5036	1:1000	Guinea pig	Peninsula; San Carlos; CA; USA
	AB 5909	1:4000	Rabbit	Millipore; Temecula; CA; USA
L-ENK	4140-0355	1:800	Mouse	Bio-Rad; Kidlington; UK
	AB5024	1:600	Rabbit	Merck; Darmstadt; Germany
nNOS	N2280	1:400	Mouse	Sigma; MSU; USA
	AB 5380	1:17000	Rabbit	Millipore; Temecula; CA; USA
NPY	NA1233	1:8000	Rabbit	Enzo Life Sciences; Farmingdale; NY; USA
	sc-133080	1:100	Mouse	Santa Cruz Biotechnology; TX; USA
PACAP	T-5039	1:300	Guinea pig	Peninsula; San Carlos; CA; USA
	T-4465	1:20000	Rabbit	Peninsula; San Carlos; CA; USA
SOM	11180	1:30	Rabbit	Icn-Cappel; Aurora; OH; USA
	T-1608	1:30	Rat	Peninsula; San Carlos; CA; USA
SP	8450-0505	1:100	Rat	Bio-Rad; Kidlington; UK
VAChT	H-V006	1:6000	Rabbit	Phoenix Pharmaceuticals Inc; Burlingame; CA; USA
VIP	VA 1285	1:6000	Rabbit	Enzo Life Sciences; Farmingdale; NY; USA
	T-5030	1:1000	Guinea pig	Peninsula; San Carlos; CA; USA
Secondary reagents				
Biotinylated anti-rabbit immunoglobulins	E 0432	1:800	Goat	Dako; Hamburg; Germany
CY3-conjugated streptavidin	711-165-152	1:8000	-	Jackson I.R.; West Grove; PA; USA
FITC-conjugated anti-mouse IgG	715-096-151	1:400	Donkey	Jackson I.R.; West Grove; PA; USA
FITC-conjugated anti-rat IgG	712-095-153	1:400	Donkey	Jackson I.R.; West Grove; PA; USA
FITC-conjugated anti-guinea pig IgG	706-095-148	1:600	Donkey	Jackson I.R.; West Grove; PA; USA

**Table 6:** List of antigens used in pre-absorption test.

Antigens Used in Pre-Absorption Test		
CGRP	C0292	Sigma; MSU; USA
DβH-blocking peptide	MBS9218238	MyBioSource; CA; USA
GAL	G5773	Sigma; MSU; USA
L-ENK	ab142314	Abcam; UK
nNOS	N3033	Sigma; MSU; USA
NPY	N3266	Sigma; MSU; USA
PACAP	A9808	Sigma; MSU; USA
SOM	S9129	Sigma; MSU; USA
SP	S6883	Sigma; MSU; USA
VAChT	V007	Phoenix Pharmaceuticals Inc; CA; USA
VIP	V6130	Sigma; MSU; USA
